# Age-associated telomere shortening in mouse oocytes

**DOI:** 10.1186/1477-7827-11-108

**Published:** 2013-11-21

**Authors:** Tomoko Yamada-Fukunaga, Mitsutoshi Yamada, Toshio Hamatani, Nana Chikazawa, Seiji Ogawa, Hidenori Akutsu, Takumi Miura, Kenji Miyado, Juan J Tarín, Naoaki Kuji, Akihiro Umezawa, Yasunori Yoshimura

**Affiliations:** 1 Department of Obstetrics and Gynecology, Keio University School of Medicine, 35 Shinanomachi Shinjuku-ku, Tokyo 160-8582, Japan; 2 Department of Reproductive Biology, National Research Institute for Child Health and Development, 2-10-1 Ohkura Setagaya-ku, Tokyo 157-8535, Japan; 3 Department of Functional Biology and Physical Anthropology, Faculty of Biological Sciences, University of Valencia, Burjassot, Valencia, Spain

**Keywords:** Reproductive aging, Postovulatory aging, Tert, Oxidative stress, Mouse oocyte, Telomere

## Abstract

**Background:**

Oocytes may undergo two types of aging. The first is induced by exposure to an aged ovarian microenvironment before being ovulated, known as ‘reproductive or maternal aging’, and the second by either a prolonged stay in the oviduct before fertilization or *in vitro* aging prior to insemination, known as ‘postovulatory aging’. However, the molecular mechanisms underlying these aging processes remain to be elucidated. As telomere shortening in cultured somatic cells triggers replicative senescence, telomere shortening in oocytes during reproductive and postovulatory aging may predict developmental competence. This study aimed to ascertain the mechanisms underlying altered telomere biology in mouse oocytes during reproductive and postovulatory aging.

**Methods:**

We studied *Tert* expression patterns, telomerase activity, cytosolic reactive oxygen species (ROS) production, and telomere length in fresh oocytes from young versus reproductively-aged female mice retrieved from oviducts at 14 h post-human chorionic gonadotropin (hCG), *in vivo* or *in vitro* postovulatory-aged mouse oocytes at 23 h post-hCG. Oocytes were collected from super-ovulated C57BL/6 J mice of 6–8 weeks or 42–48 weeks of age. mRNA and protein expressions of the *Tert* gene were quantified using real-time quantitative reverse transcriptase polymerase chain reaction (Q-PCR) and immunochemistry. Telomerase activity was measured by a telomeric repeat amplification protocol assay, while telomere length was measured by Q-PCR and quantitative fluorescence in situ hybridization analyses.

**Results:**

The abundance of *Tert* expression in oocytes significantly decreased during reproductive and postovulatory aging. Immunofluorescent staining clearly demonstrated an altered pattern and intensity of TERT protein expression in oocytes during reproductive aging. Furthermore, relative telomerase activity (RTA) in oocytes from reproductively-aged females was significantly lower than that in oocytes from young females. In contrast, RTA in postovulatory-aged oocytes was similar to that in fresh oocytes. Oocytes from reproductively-aged females and postovulatory-aged oocytes showed higher ROS levels than oocytes from young females. Relative telomere length (RTL) was remarkably shorter in oocytes from reproductively-aged females compared to oocytes from young females. However, postovulatory aging had no significant effect on RTL of oocytes.

**Conclusions:**

Long-term adverse effects of low telomerase activity and increased ROS exposure are likely associated with telomere shortening in oocytes from reproductively-aged female mice.

## Background

It is well known that fertility in women begins to decline by their mid-30s and pregnancies in older women result in higher rates of miscarriage and/or aneuploid offspring [1]. Extensive clinical experience with oocyte donation to women of advanced age in the USA revealed that embryos generated from oocytes of young women could be implanted efficiently in women above the age of 40 or even postmenopausal. This finding suggests that a decrease in oocyte quality contributes to the drop in fertility as women age. On the other hand, women are now marrying later and consequently, the average age of women when they first attempt childbearing has increased. The reproductive-capacity decline in oocyte quality with aging has therefore risen in importance with respect to human reproduction. However, the molecular events involved in this process remain poorly understood.

The relevant theory for ovarian aging implies a reduced ability of oocytes to counteract ROS, which are among the most important physiological inducers of cellular injury associated with aging [2]. Mitochondria are both the major cellular source of ROS and one of the main targets of ROS-induced oxidative damage [3], as well as playing an important role in cell death via apoptosis [4]. Thus, mitochondrial dysfunction and oxidative stress have been implicated in cellular senescence and aging [5]. With respect to ovarian aging, increased ROS may partially contribute to follicular atresia and to the decline in number and quality of oocytes [6]. Exposure of mouse oocytes to H_2_O_2_ (200 μM) completely inhibits cleavage to two-cell-stage embryos [7], while endogenous H_2_O_2_ levels in human embryos correlate with apoptotic activity [3]. Mitochondrial dysfunction could also compromise developmental processes by inducing chromosomal segregation disorders, maturation and fertilization failures, or oocyte/embryo fragmentation resulting in mitochondria-driven apoptosis [8]. Collectively, mitochondrial dysfunction and oxidative stress are clearly important contributors to oocyte aging in ovaries.

Oocytes undergo two aging phenomena. The exposure of oocytes to an aged ovarian microenvironment is responsible for a female age-dependent process known as ‘reproductive aging’ [6] or ‘maternal aging’. On the other hand, a prolonged stay in the oviduct before fertilization or *in vitro* culture prior to insemination involves a time-dependent aging process, known as ‘postovulatory aging’ [9]. *In vivo* postovulatory aging of oocytes, when they remain unfertilized in the oviduct for a prolonged time after ovulation, is known to significantly affect the development of mammalian oocytes [10]. Many *in vivo* studies have shown that such postovulatory aging frequently results in lower fertilization percentages [11], with the limit for optimal fertilization determined in mouse (8 – 12 h) and human (24 h) [12]. On the other hand, *in vitro* postovulatory aging of oocytes, via prolonged *in vitro* culture of oocytes before fertilization, is a clinical issue of increasing importance. Indeed, some investigators have proposed “rescue” intracytoplasmic sperm injection (ICSI) for oocytes that fail to fertilize during insemination. Rescue ICSI at 6 h post-insemination (46 h post-hCG) gives better fertilization rates; however, pregnancy and implantation rates decrease with rescue ICSI at 22 h post-insemination when oocytes are aged [13].

The current study used two mouse models, for reproductive aging and postovulatory aging, to explore the molecular mechanisms underlying impaired developmental competence in oocytes. Both aging processes induce similar alterations in oocytes, such as metaphase II aberrations, spontaneous activation, cellular fragmentation, and initiation of an apoptotic pathway, and lead to faulty spindle checkpoints, which predispose oocytes to premature chromosome separation and aneuploidy [14]. Most importantly, both maternal and postovulatory aging of oocytes involve a decline in mitochondrial function and changes in the redox state [9,15]. Takahashi *et al*. [16] reported that oxidative stress was significantly increased in oocytes aged *in vivo* compared to fresh control oocytes, while Tatone *et al*. [9] showed increased ROS levels in oocytes aged *in vitro* after ovulation as well as in oocytes from reproductively-aged females, compared with fresh oocytes from young females mice. Thus, ROS seemingly plays an important role in both the maternal and postovulatory aging process in oocytes [17,18].

Microarray analysis revealed altered gene expression patterns in oocytes during reproductive aging [19], although the genes altered are associated with chromatin structure, DNA methylation, genome stability, and RNA helicases, which is unique to aging in oocytes compared with aging in somatic cells and organs. Despite this, the generally accepted view of aging as described above is also presented, including expression changes of genes involved in mitochondrial function (e.g., *Sdha,* known to be an index for mitochondrial activity, and *Pdhb*) and response to oxidative stress (e.g., *Sod1* and the thioredoxin family genes such as *Txn1* and *Apacd*). Furthermore, telomere reverse transcriptase (*Tert*) is more highly expressed in oocytes from young females. The *Tert* mRNA codifies for the catalytic component (TERT) of telomerase, with the other enzyme component an RNA template (TERC) [20]. Both components constitute active telomerase, which compensates for the progressive shortening of chromosomes with each round of DNA replication by maintaining the telomeric DNA sequences [21].

Telomere shortening is characterized by cell cycle arrest and apoptosis in cultured somatic cells showing low telomerase expression and activity that approaches the “Hayflick Limit” trigger of replicative senescence [22]. Reduced telomerase activity also plays an integral role in granulosa cell apoptosis and follicular atresia [23]. Although mouse telomeres are substantially longer overall than human telomeres, mouse ovaries have reduced telomerase activity and telomere length during reproductive aging [24,25]. Oxidative stress can also cause telomere shortening because the triple-G-containing telomeres structure is highly sensitive to oxidative damage [26]. When the protonophore carbonyl cynide p-trifluoromethoxyphenylhydrazone (FCCP) is used at 750 nM to uncouple mitochondrial electron transport and disrupt mitochondrial function in 1-cell zygotes, ROS is dramatically induced within 20 min in the embryos and telomeres are significantly shortened at the 2-cell stage within 24 hours after FCCP treatment [27]. The mechanism controlling oocyte telomere length is relatively unknown. Turner *et al*. analyzed female age as a possible factor related to human oocyte telomere length, but found no association [28]. Mice engineered to have short telomeres display problems with fertility and embryo development [29]. However, despite these data, little evidence has emerged to explain changes in telomerase activity and telomere length during aging in oocytes. To this end, the current study investigated the mechanisms underlying altered telomere biology in mouse oocytes during female and postovulatory aging.

## Methods

### Collection and manipulation of oocytes

The current study assumed that oocytes from C57BL/6 J mice at 12 months of age provide an appropriate model for female reproductive aging, because the reproductive lifespan of this strain ends earlier than that of other mouse strains, including the Kunming outbred mice from ICR [28]. Quantitative cytological analyses of aging C57BL/6 J mouse ovaries revealed that primordial and growing follicles are nearly exhausted by 13–14 months [24]. C57BL/6 J mice are reported to produce very small litters (6–7 pups) in their youth and no pups by 12 months of age [24]. Therefore we used female C57BL/6 J mice at 42–48 weeks of age for this study.

Female C57BL/6 J mice were superovulated at 6–8 weeks (young females) or 42–48 weeks of age (reproductively-aged females) by injection of 5 IU of pregnant mare serum gonadotropin (PMSG, Sigma-Aldrich, St Louis, MO, USA), followed 46–48 hours later by 5 IU of human chorionic gonadotropin (hCG, Sigma-Aldrich). Fresh oocytes retrieved from either young or reproductively-aged females were harvested 14 h after the hCG injection by a standard published method [30].

**Figure 1:**
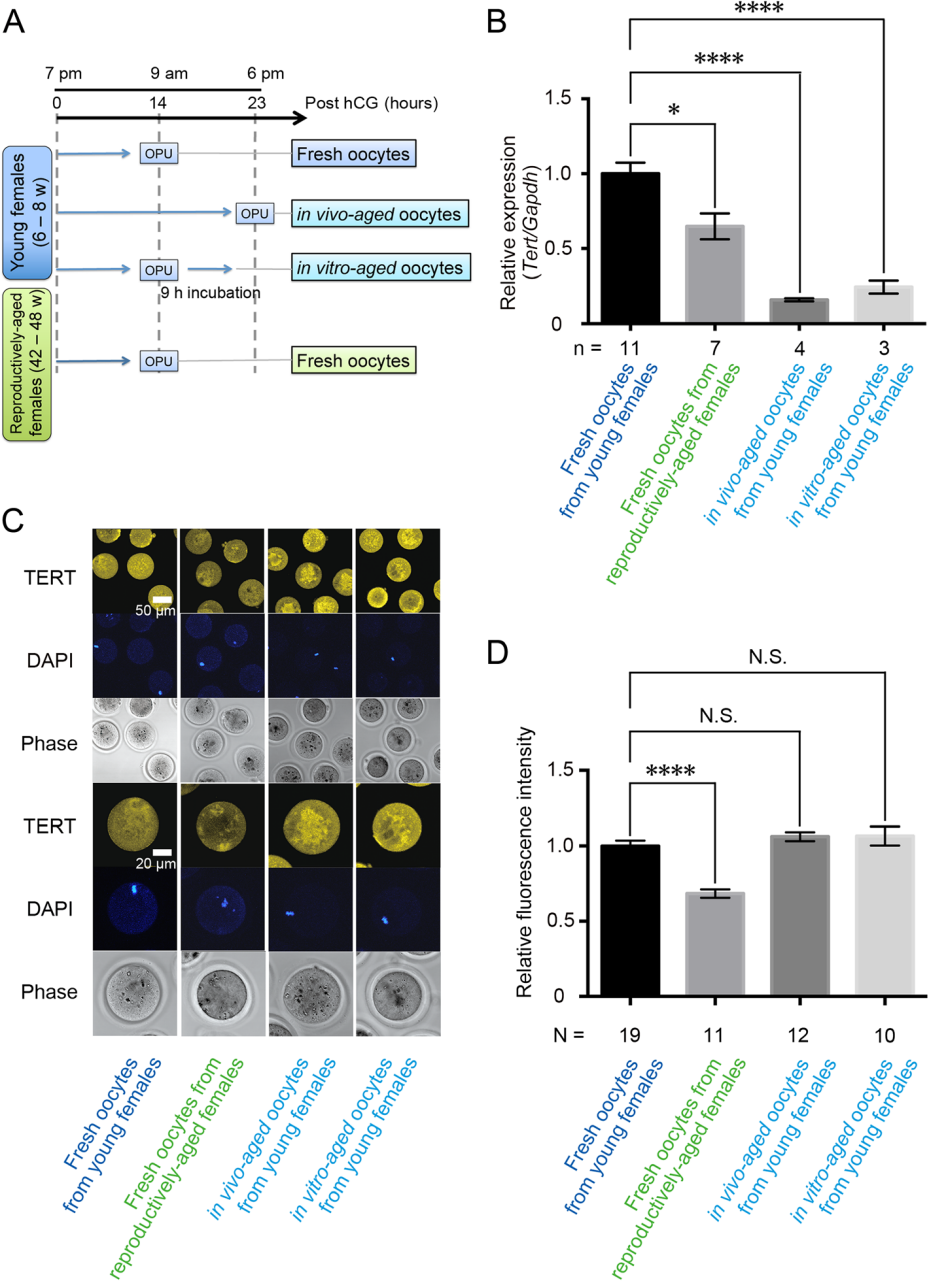
**Scheme of MII oocyte collection and expression analysis of Tert transcripts and protein. (A)** Diagram showing the study design for collecting fresh oocyte retrieved from young or reproductively-aged females, and oocytes aged *in vivo* or *in vitro* after ovulation (OPU: oocyte pick up). **(B)** Q-PCR analysis of *Tert* in MII oocytes. *Gapdh* was used as an internal standard for gene expression (n: number of replicated experiments). The error bars show mean ± SEM. *P < 0.05; ****P < 0.0001. **(C)** Immunocytochemical analysis of TERT expression (yellow). Nuclei were stained with DAPI (blue). Fresh oocytes from reproductively-aged females exhibited weaker TERT signal intensity than fresh oocytes from young females. **(D)** Quantification of relative TERT fluorescence intensities shown in 1C (N: total number of oocytes). The error bars show mean ± SEM. ****P < 0.0001.

Postovulatory oocytes were subclassified into *in vivo* and *in vitro-*aged oocytes. *In vivo* postovulatory-aged oocytes were recovered from young superovulated mice of 6–8 weeks of age at 23 hours after hCG. Following the removal of cumulus cells by incubation in M2 medium (EmbryoMax M2 Medium Powdered with phenol red; Millipore, Billerica, MA, USA) containing 300 μg/ml hyaluronidase (Sigma-Aldrich), oocytes were washed thoroughly in phosphate-buffered saline (PBS) supplemented with 3 mg/ml polyvinylpyrrolidone (PVP), and then selected based on good morphology, in particular, the presence of a clear and moderately granular cytoplasm, small perivitelline space, intact first polar body, colorless zona pellucida, and no fragmentation via conventional phase-contrast microscopy [31]. *In vitro-*aged oocytes were retrieved from young superovulated mice at 14 hours post-hCG, subjected to cumulus cell removal as described above, and then incubated in synthetic oviductal medium enriched with potassium (EmbryoMax KSOM Powdered Mouse Embryo Culture Medium; Millipore, Billerica, MA, USA) at 37°C in an atmosphere of 95% air/5% CO_2_ for 9 hours before washing and collection (Figure 1A).

### Ethical approval for the use of animals

All experiments were conducted in accordance with the Laboratory Animal Care and Use Committee of Keio University School of Medicine (approval number #10240-1838).

### RNA extraction and real-time quantitative reverse transcriptase polymerase chain reaction (Q-PCR)

More than three subsets of 10 oocytes were collected for Q-PCR as described above, transferred into PBS supplemented with 3 mg/ml PVP, and stored in liquid nitrogen. Total RNA was extracted using a PicoPure RNA Isolation Kit (Arcturus, La Jolla, CA, USA) and reverse transcribed in a 20 μl volume using poly-A primer and Superscript III reverse transcriptase (Invitrogen Carlsbad, CA, USA) according to the manufacturer’s instructions. The resultant cDNA was subjected to Q-PCR using the SYBR Green Realtime PCR Master Mix (Toyobo, Osaka, Japan) and ABI Prism 7700 Sequence Detection System (Applied BioSystems, Framingham, MA, USA) as described previously [19,32]. An amount of cDNA equivalent to 1/2 oocyte was used for each real-time PCR reaction with a minimum of three replicates, and with no-enzyme and no-template controls for each gene. Data were normalized against *Gapdh* by the Ct method [32]. PCR primers used for the *Tert* and *Gapdh* genes are listed in Additional file 1: Table S1. Calculations were automatically performed by ABI software (Applied BioSystems).

### Immunocytochemistry of oocytes

Samples for immunocytochemical staining were fixed in 4% paraformaldehyde (Wako Pure Chemical, Osaka, Japan) with 0.1% glutaraldehyde (Wako) in PBS for 10 min at room temperature (RT), and then permeabilized with 0.5% Triton X-100 (Sigma-Aldrich) in PBS for 30 min. The fixed samples were incubated at RT with primary antibody for 60 min, followed by the relevant secondary antibody for 60 min. The anti-TERT antibody (Santa Cruz Biotechnology, Santa Cruz, CA, USA) was used at 1: 50 dilution, followed by Alexa Fluor 633 goat anti-rabbit IgG (Invitrogen) as the secondary antibody. The cellular DNA (nuclei) was stained with 4′, 6-diamidino-2-phenylindole (DAPI; Wako; diluted 1: 300). The cells were then washed with PBS and viewed by laser confocal microscopy (LSM710, Zeiss, Carl Zeiss Oberkochen, Germany). TERT signal intensities of each oocytes were measured using ImageJ software (version 1.47: National Institutes of Health, Bethesda, MD).

### Telomerase activity measurement

Telomerase activity was measured by a PCR-based telomeric repeat amplification protocol (TRAP) assay using a commercial kit (The TELO TAGGG PCR ELISA Telomerase PCR ELISA kit; Roche Molecular Biochemicals, Mannheim, Germany) according to the manufacturer’s instruction and as described previously [33]. More than three subsets of 50 oocytes were analyzed. To collect each subset of oocytes, 5–10 mice were sacrificed and used for experiments. More than three replicated experiments were carried out, in which each sample was assayed at least three times and an average value was taken. The level of telomerase activity in each sample was determined by signal comparison against a known amount of a control template. Relative telomerase activity (RTA) of each sample was calculated as described previously [33].

### Detection of cytosolic ROS production

Rates of cytosolic ROS generation in oocytes were measured using dihydroethidium (HEt) (Invitrogen), a nonfluorescent derivative of ethidium that is oxidized to a fluorescent product by superoxide. Oocytes were incubated in M2 medium containing 50 μM HEt for 15 min [34] and viewed by laser confocal microscopy (LSM710, Zeiss). The experiment was repeated three times.

### Telomere measurement

Q-PCR assay enables to measure telomere length from small amounts of gDNA. Callicott and Womack [35] reported that the results from real-time qPCR assays of mouse DNA were similar to terminal restriction fragment (TRF) analysis by pulse-field gel electrophoresis followed by Southern hybridization, which is generally considered the most accurate technique for telomere length measurement. However, this method might be difficult for oocytes due to insufficient material for Southern blotting. Turner *et al*. also used Q-FISH to successfully measured telomere lengths in human oocytes and preimplantation embryos, and found a significantly shorter average telomere length in cleavage-stage embryos than in blastocysts or GV oocytes [36]. Their data showed that total fluorescence per nucleus was related to the number of telomere signals per nucleus, but there was no significant relationship between fluorescence and nuclear area. We thus chose to measure telomere length using Q-PCR and Q-FISH.

### Quantitative fluorescence in situ hybridization (Q-FISH)

*Preparation of slides:* Oocytes were retrieved from three mice per experiment, and three oocytes were selected for good morphology. These oocytes were placed into a drop of hypotonic solution (1% sodium citrate and 0.5% bovine serum albumin) to induce swelling for clearer visualization of the nucleus, followed by fixation in methanol: acetic acid (3: 1). These fixed samples were applied in a drop onto a glass slide and slides were air dried at RT. The experiment was repeated twenty one times in total.

*Telomere Q-FISH:* Q-FISH was performed according to the protocol provided by the manufacturer of the peptide nucleic acid (PNA) probe (Telomere PNA FISH kit/Cy3, DAKO, Glostrup, Denmark) with minor modifications.

*Image analysis:* Telomeres and chromosomes in the samples were observed by laser confocal microscopy (LSM710, Zeiss) using a Cy3-DAPI filter set. Telomere images were exported as 8-bit tagged image file format (tiff) files and analyzed using the TFL-telo program [36,37]. Fluorescent densities of individual dots were measured to calculate average densities.

### Telomere measurement by quantitative real-time PCR

More than three subsets of 20 oocytes were collected. For each subset, 5 young mice and 10 reproductively-aged mice were sacrificed and used for experiments. Oocytes were washed in PBS-PVP, and then stored at -20°C until subsequent DNA extraction using a QIAmp DNA micro Kit (Qiagen, Valencia, CA, USA) and quantification. Average telomere length was measured from the total genomic DNA using Q-PCR as previously described [19,32]. PCR reactions were performed on the ABI Prism 7700 Sequence Detection System (Applied Biosystems), using telomeric primers, primers for the reference control gene (mouse 36B4 single copy gene), and PCR settings as previously described [35]. The telomere signal was normalized to the signal from the single-copy gene to generate a relative telomere to single copy gene (T/S) ratio indicative of relative telomere length. Equal amounts of DNA (300 pg) were used for each reaction with several repeats.

### Statistical analysis

A one-way analysis of variance (ANOVA) with Bonferroni’s multiple comparison testing was used for statistical analysis. Qualitative variables of HEt-positive oocytes were compared with a chi-square test, using 95% confidence intervals. To counter the inflated Type I error, we adopted a conservative alpha level by performing a Ryan’s adjustment. The non-parametric Kruskal–Wallis test was employed for the telomere Q-FISH analysis and comparisons of groups were performed by the Dunn test. Statistical analyses were performed using GraphPad Prism software (GraphPad, San Diego, CA, USA). P < 0.05 was considered to indicate statistical significance.

## Results

Using *Gapdh* as an internal standard for gene expression, transcript levels of *Tert* were significantly reduced in fresh oocytes from reproductively-aged females (0.65 ± 0.09) compared with those in fresh oocytes from young females (1.00 ± 0.07) (Figure 1B). Transcript levels of *Tert* of postovulatory-aged oocytes (*in vivo*: 0.16 ± 0.01 *in vitro*: 0.24 ± 0.04) were significantly decreased compared to the levels found in fresh oocytes from young females (Figure 1B). TERT expression in MII oocytes was analyzed in more detail by immunostaining with antibodies to TERT and careful observation of individual cells. TERT protein was clearly detected in the cytoplasm, but not on the chromosomes in MII oocytes from young females. Oocytes from reproductively-aged females (0.68 ± 0.03) showed significantly lower signal intensity of TERT than oocytes from young females (1.00 ± 0.04). However, there was no difference in TERT expression between *in vivo* (1.06 ± 0.03) or *in vitro-*aged oocytes from young females (1.07 ± 0.06) and fresh oocytes from young females (Figure 1C and 1D).

**Figure 2:**
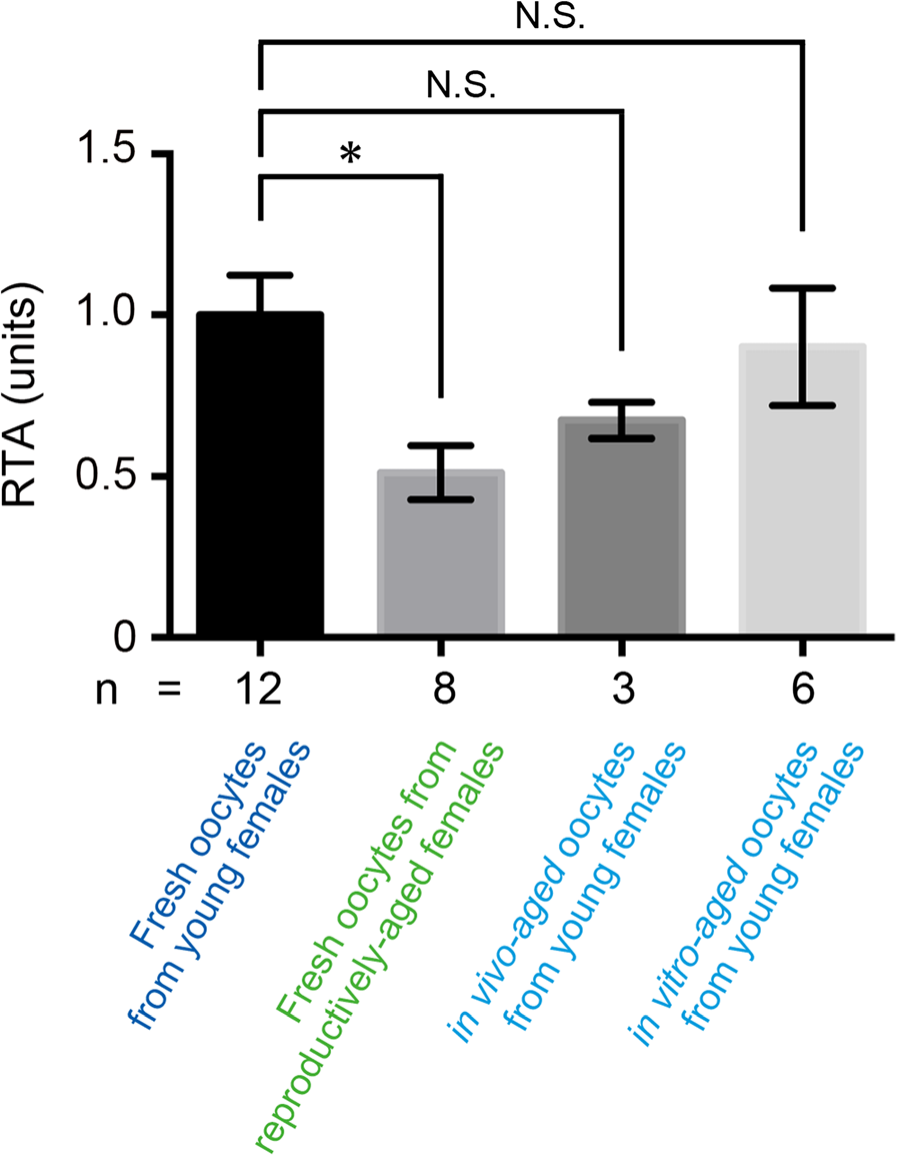
**RTA in MII oocytes during reproductive aging and postovulatory aging.** RTA was measured in fresh oocytes retrieved from young or reproductively-aged females and postovulatory-aged oocytes (n: number of replicated experiments). Each RTA was represented by a ratio of absorbance at 450 nm for oocyte samples compared with that for the corresponding known amount of a control template. The error bars show mean ± SEM. *P < 0.05.

We next investigated whether decreased TERT expression negatively affects telomerase activity. RTA in fresh oocytes from reproductively-aged females (0.51 ± 0.08) was significantly lower than that in fresh oocytes from young females (1.00 ± 0.12) (Figure 2). In contrast, RTA was not significantly different between postovulatory-aged (*in vivo*: 0.67 ± 0.06; *in vitro*: 0.90 ± 0.18) and fresh oocytes from young females (Figure 2).

**Figure 3:**
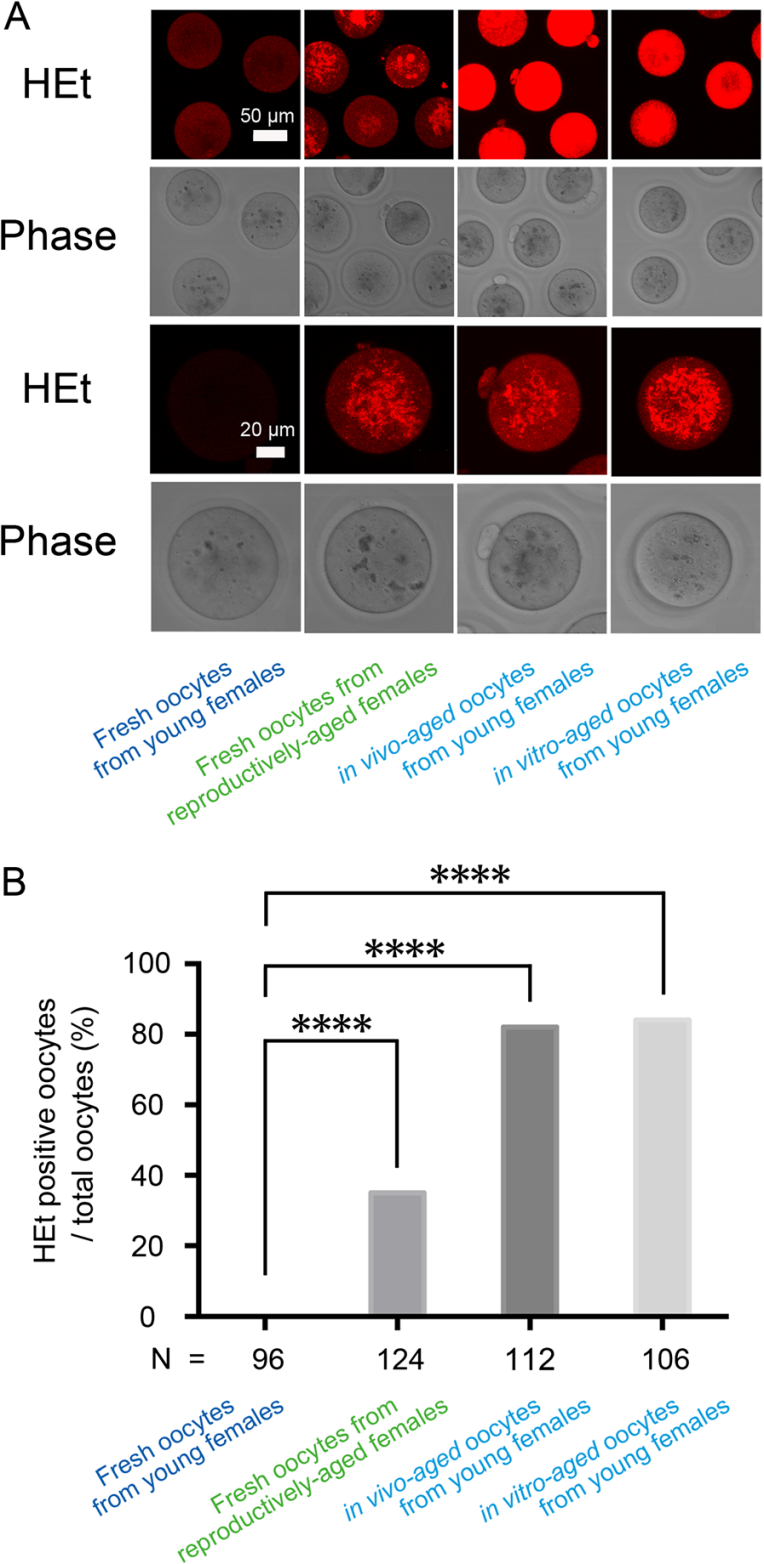
**Generation of ROS in MII oocytes during female and postovulatory aging. (A)** Fluorescent microscope images of hydroethidium (HEt)-labeled oocytes. **(B)** Percentage of HEt-positive oocytes (N: total number of oocytes analyzed). Reproductively-aged females exhibited a significantly higher percentage of HEt-positive oocytes, indicating increased ROS levels, than oocytes from young females. Furthermore, *in vivo* and *in vitro* postovulatory-aged oocytes showed significantly increased ROS levels compared to fresh oocytes from young females. ****P < 0.0001.

The regulation of intracellular redox potential is a crucial determinant of oocyte competence. We therefore measured the rate of oxidation of HEt to detect alterations in the redox state induced by oocyte aging. Fresh oocytes from reproductively-aged females and postovulatory-aged oocytes showed a significantly higher percentage of HEt-positive staining than fresh oocytes from young females. In particular, 0.0% (0/96) in fresh oocytes from young females, 35.5% (44/124) in reproductively-aged oocytes, 82.1% (92/112) in *in vivo-*aged oocytes, and 84.9% (90/106) in *in vitro*-aged oocytes from young females exhibited HEt-positive staining (Figure 3A and 3B).

**Figure 4:**
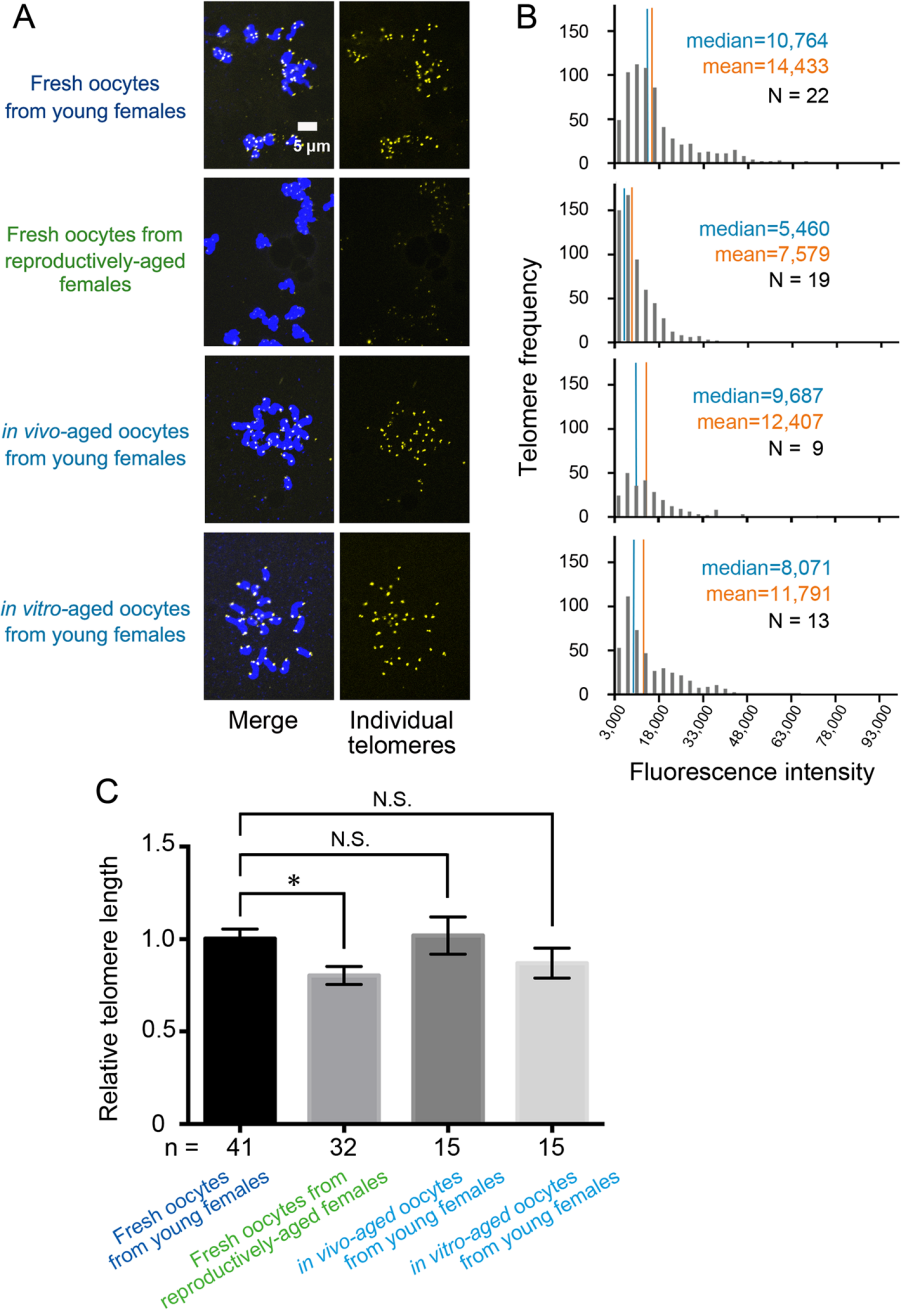
**Telomere length in MII oocytes during female and postovulatory aging. (A)** Telomere Q-FISH images of oocytes. Blue: DAPI-stained chromosomes, Yellow: telomeres. **(B)** Frequency distributions of telomere fluorescence in metaphase spreads of fresh oocytes from young females (telomere numbers = 695), fresh oocytes from reproductively-aged females (telomere numbers = 576), *in vivo-*aged (telomere numbers = 244), and *in vitro*-aged (telomere numbers = 444) oocytes. Blue lines in the histograms indicate medians of the distribution. Yellow lines show means (N: total number of oocytes). **(C)** Relative telomere length shown as the T/S ratio by qPCR analysis. The error bars show mean ± SEM. *P < 0.05 (n: represents number of replicated experiments).

To determine how decreased telomerase activity might affect telomere length, we conducted Q-FISH and Q-PCR analyses. Q-FISH showed stronger telomere signal intensities in fresh oocytes from young females than in oocytes from reproductively-aged females. In contrast, signal intensities were almost identical between *in vivo* or *in vitro* postovulatory-aged and fresh oocytes from young females (Figure 4A, and Additional file 2: Figure S1). The frequency distributions of telomere fluorescence metaphase spreads indicated longer telomeres in oocytes from young females (median intensity = 10,764) than in oocytes from reproductively-aged females (median intensity = 5,460). There was no significant difference between postovulatory-aged oocyte groups (*in vivo:* median intensity = 9,687, *in vitro:* median intensity = 8,071) (Figure 4B). In addition, measurement of relative telomere length expressed as a T/S ratio by Q-PCR revealed shorter telomeres in oocytes from reproductively-aged females (0.80 ± 0.05) compared to those in oocytes from young females (1.00 ± 0.05). The telomere length in *in vivo-*aged oocytes (1.02 ± 0.10) and *in vitro-*aged oocytes from young females (0.87 ± 0.08) was not significantly different from that evidenced in fresh oocytes from young females (Figure 4C).

## Discussion

### Oocytes from reproductively-aged female mice display telomere shortening

The present study shows a significant decrease in the abundance of *Tert* transcripts and protein as well as telomerase activity and telomere shortening in mouse oocytes retrieved from reproductively-aged females when compared to oocytes from young females. It is known that cytoplasmic fragmentation, a reliable morphological marker of apoptosis used to predict developmental potential in human preimplantation embryos [38], increases with reproductive aging. In addition, telomere shortening has been recently shown to predict cytoplasmic fragmentation in early-stage embryos [39]. The current study was focused only on MII mouse oocytes showing good general morphology and no fragmentation. Nevertheless, the Q-PCR and Q-FISH data demonstrated that reproductive aging is associated with shorter telomere lengths even in mouse oocytes that retained these healthy morphological features.

### Decreased TERT expression and telomerase activity may contribute to telomere shortening in oocytes from reproductively-aged female mice

There are three possible molecular mechanisms responsible for telomere shortening in oocytes from reproductively-aged females: (*1*) replicative senescence, which is the normal shortening resulting from chromosomal replication [20]; (*2*) loss of telomerase activity [40]; and/or (*3*) damage by ROS [41].

Replicative senescence is unlikely to occur in oocytes after they enter into meiosis. Nonetheless, primordial cells and oogonia may display replicative senescence when actively proliferate during fetal life. As oogonia enter into meiosis during this period of time, the resulting putative replicative senescence would affect oocytes in both young and reproductively-aged female groups. This is not the case, however, for mitotically active oogonial stem cells (OSCs) that are present in the surface ovarian epithelium and contribute to follicular renewal and oogenesis during postnatal life in mammals [42]. As OSCs are continually dividing during female’s reproductive life, OSCs and the resulting oocytes in aged females may exhibit higher replicative senescence and telomere shortening compared to OSCs and oocytes in young females.

In the present study, oocytes from reproductively-aged female mice showed a significant decline in TERT expression and telomerase activity compared to oocytes from young females. Some of the oocytes from young females also showed subcortical and perinuclear patterns of TERT expression, while others showed a more homogeneous pattern. However, oocytes from all aged females showed significantly diminished TERT expression and a more random pattern. TERT is a catalytic subunit of the enzyme telomerase. Telomerase-null mice lacking telomerase activity undergo progressive telomere shortening over generations, resulting in abnormal spindles and misalignment of metaphase chromosomes in oocytes, with female sterility the eventual outcome [43]. Phenotypic analyses of telomerase-null mice suggested that germ cells are more susceptible to lack telomerase activity than somatic cells. Chromosome misalignment and disruption of meiotic spindles at the metaphase stage appeared frequently in oocytes from fourth-generation telomerase knockout mice, while meiotic progression, chromosome behavior, and spindle morphology in first-generation telomerase-null mouse oocytes were comparable to those of wild-type mouse oocytes [44]. Since spindle formation is a critical event in proper chromosome segregation, this observation implicated telomere involvement in the development of oocyte aneuploidy in oocytes from reproductively-aged females. Although no studies have yet determined the extent and acceleration of chromosomal abnormalities in oocytes from reproductively-aged telomerase-null mice, these findings support the decline in TERT expression and activity in mouse oocytes with reproductive aging and its association with telomere shortening and decline in oocyte quality [39].

Keefe *et al*. [39] studied sibling MII human oocytes and embryos and found that the telomere lengths between sibling oocytes ranged widely (0.5–24.5 kb). Thus, it was not possible to assume similar telomere lengths between sibling oocytes in the present study. In addition, Turner *et al*. [28] reported no association between female age and human oocyte telomere length, although their data still showed extensive variability in telomere lengths for sibling MII oocytes (6–14 kb). In the current study, telomere length in mouse MII oocytes was measured with Q-PCR and Q-FISH. Since mouse telomeres are substantially longer overall than human telomeres, the mouse models we used to study telomere biology in oocyte aging do not necessarily represent that same process in human oocytes. Although extensive studies are needed to clarify this issue, the long-term adverse effects of low telomerase activity and increased ROS exposure during reproductive aging likely lead to telomere shortening in mouse oocytes, which in turn may contribute to the reported age-associated impairment of developmental competence.

### Postovulatory-aged oocytes did not show significant changes in telomerase activity and telomere length

Unlike reproductive aging, *in vivo* and *in vitro* postovulatory aging was not associated with decreased TERT protein expression or telomerase activity in oocytes. The telomere length analyses by Q-PCR and Q-FISH also showed no telomere shortening in postovulatory-aged oocytes. These results could be attributed to the short time (9 hours) of oxidative stress loading during postovulatory aging used in this study. For example, it has been reported that whereas zygotes do not show telomere shortening at 6 hours after the FCCP treatment, 2-cell embryos show significant telomere shortening at 24 hours after FCCP treatment [27]. Alternatively, the adverse effect of long-term ROS loading on telomere length during reproductive aging may be enhanced by other factors including low telomerase activity. Furthermore, it is possible that different molecular mechanisms underlie reproductive and postovulatory aging of oocytes. For example, mitochondria are one of the main targets and source of ROS-induced oxidative damage. Mitochondrial damage could directly compromise developmental processes by fertilization failures or embryo fragmentation resulting in mitochondria-driven apoptosis [8]. It is also reported that mutations in mtDNA have been found in oocytes from older women [45], in which mitochondrial dysfunction in turn would lead to increased ROS. Although reproductive aging is associated with a significant decrease in number and function of mitochondria in oocytes [46,47], mitochondrial damage in oocytes with postovulatory aging could be much more severe than that experienced with reproductive aging. In fact, Takahashi *et al*. demonstrated that calcium oscillations at fertilization become lower in amplitude and higher in frequency in postovulatory-aged oocytes, but do not significantly change in fresh oocytes from reproductively-aged females, compared to those in fresh oocytes from young mice [15,16,48].

### ROS contributes to telomere shortening in oocytes from reproductively-aged female mice but not in postovulatory-aged oocytes

A higher percentage of fresh oocytes from reproductively-aged females displayed HEt-positive fluorescence when compared to fresh oocytes from young mice. The ROS generated by compromised mitochondria could potentially oxidize proteins necessary for telomere maintenance, resulting in genomic instability [27]. Furthermore, telomeres are especially susceptible to ROS-induced DNA damage, because their sequence is guanine rich and the nucleotide is most susceptible to oxidation. Telomeres also sit in the nuclear membrane, where they are susceptible to lipid peroxidation and lack protective proteins [41]. Direct administration of oxidants to cells is known to damage DNA, break polyguanosine sequences in telomere repeats, and cause telomere shortening, cell cycle arrest, and replicative senescence [26]. With respect to ovarian aging, but not oocyte aging, telomerase activity declines in mouse ovaries with age [28]. In turn, the anti-oxidant, N-acetyl-L-cysteine (NAC), prevents ROS-induced damage and loss of telomerase activity in aging mouse ovaries [28]. Another study reported an adverse effect of ROS on preimplantation development [27], wherein one-cell mouse zygotes treated with FCCP, which uncouples the mitochondrial electron transport pathway, showed significantly decreased developmental competence with telomere shortening compared to control embryos. Increased ROS together with telomere shortening/loss, chromosomal fusion, and apoptosis were observed in these FCCP-treated embryos [27]. All these findings support the notion that ROS can cause telomere shortening in oocytes from reproductively-aged mice. However, whereas in the current study *in vivo* and *in vitro* postovulatory-aged oocytes from young females showed significantly higher ROS levels than oocytes from reproductively-aged females, oocytes did not show telomere shortening. Therefore, in oocytes from reproductively-aged females telomere shortening is associated not only with high ROS levels but also with low telomerase activity. In contrast, in postovulatory-aged oocytes any of these factors is associated with telomere shortening although ROS-induced telomere shortening may need longer periods of time to manifest. Alternatively, molecular mechanisms other than ROS and telomere dysfunction may contribute to deficiencies in postovulatory aging.

### Telomere shortening may contribute to the decline in developmental competence of oocytes with reproductive aging

Telomere dysfunction has been linked to many aspects of the aging process including chiasmata, synapsis, meiosis, and impaired egg quality by promoting aneuploidy in mammals. Recently, Sahin *et al.* reported that telomere dysfunction-induced activation of p53 represses proliferator-activated receptor gamma coactivator 1 (PGC-1), thereby linking telomeres to mitochondrial biology, oxidative defense, and metabolism [49]. FCCP-induced telomere shortening leads to Robertosonian fusions of chromosomes and genomic instability in preimplantation embryos [27]. Furthermore, the association of telomere shortening with aneuploidy has been demonstrated in human oocytes and early preimplantation embryos, but not in embryos at the blastocyst stage [50]. This circumstance may indicate that telomeres are normalized between the cleavage and blastocyst stages of embryogenesis and many embryos with short telomeres arrest during preimplantation development [50]. All this information suggests that telomere dysfunction may cause inappropriate chromosome segregation during human oocyte cell division (meiosis) and may serve as a marker for oocytes and embryos that lack the ability to produce healthy children.

At last but very important, recent studies have suggested novel roles of TERT in several essential cell signaling pathways without apparent involvement of its well-established function in telomere maintenance. For example, TERT may have a role in regulating oxidative damage-induced apoptosis in mitochondria or as a transcriptional modulator of the Wnt-beta-catenin signaling pathway [51]. Therefore, any alternative function of TERT other than telomere maintenance may explain the causality between decreased expression of TERT and the decline in developmental competence in oocytes from reproductively-aged females.

## Conclusions

Collectively, telomere shortening was observed in oocytes from reproductively-aged female mice, but not in postovulatory-aged oocytes. Although both aging processes were associated with oxidative stress, different molecular mechanisms seem to be at play. Long-term adverse effects of low telomerase activity and increased ROS exposure during reproductive aging are likely associated with telomere shortening in oocytes from reproductively-aged females.

## Abbreviations

ANOVA: Analysis of variance; DAPI: 4′, 6-diamidino-2-phenylindole; FCCP: Protonophore carbonyl cynide p-trifluoromethoxyphenylhydrazone; hCG: Human chorionic gonadotropin; HEt: Dihydroethidium; ICSI: Intracytoplasmic sperm injection; NAC: N-acetyl-L-cysteine; OPU: Oocyte pick up; OSCs: Oogonial stem cells; PBS: Phosphate-buffered saline; PGC-1: Proliferator-activated receptor gamma coactivator 1; PMSG: Pregnant mare serum gonadotropin; PNA: Peptide nucleic acid; PVP: Polyvinylpyrrolidone; Q-PCR: Real-time quantitative reverse transcriptase polymerase chain reaction; Q-FISH analysis: Quantitative fluorescence in situ hybridization analysis; ROS: Reactive oxygen species; RT: Room temperature; RTA: Relative Telomerase Activity; RTL: Relative telomere length; TERC: Template RNA component; TERT: Telomere reverse transcriptase; TRAP assay: PCR-based telomeric repeat amplification protocol assay; TRF: Terminal restriction fragment; T/S ratio: Relative telomere to single copy gene ratio.

## Competing interests

The authors declare that there is no conflict of interest that would prejudice the impartiality of the scientific work.

## Authors’ contributions

TYF, MY and TH were involved in the experimental design, the data acquisition, analysis, interpretation and drafting of the article. NC, SO, HA, TM and NK provided technical support and were involved in data analysis and interpretation. AU, KM, JJT and YY assisted with experimental design and were involved in data analysis and interpretation. All authors read and approved the final manuscript.

## Supplementary Material

Additional file 1: Table S1 Primer sets used in the current study. Click here for file

Additional file 2: Figure S1 Comparison of average telomere fluorescence of oocytes from young females, oocytes from reproductively-aged females, and *in vivo*-aged and *in vitro*-aged oocytes from young females. Box plots represent the median and inter-quartile ranges, with whiskers representing the 5th and 95th percentiles, respectively. *P < 0.05 (N: total number of oocytes). Click here for file
