# Supplementary material for: Age-associated telomere shortening in mouse oocytes
Source: Reprod Biol Endocrinol. 2013 Nov 21;11:108. doi: 10.1186/1477-7827-11-108 (PMC3842639; doi:10.1186/1477-7827-11-108)
Supplement: Additional file 1: Table S1 — Primer sets used in the current study. [file 1477-7827-11-108-S1.doc]

**Table S1** Primer sets used in the current study

| Products | Primer sets |
| --- | --- |
| Telomere | 5′-CGGTTTGTTTGGGTTTGGGTTTGGGTTTGGGTTTGGGTT-3′ |
|  | 5′-GGCTTGCCTTACCCTTACCCTTACCCTTACCCTTACCCT-3′ |
| 36B4 | 5′-GTTGGGAGTTGGACTATGGAC-3′ |
|  | 5′-TGAACTGATTGGACACACACA-3′ |
| Tert | 5′-TGGGGCCCGAGGGCAGGCGG-3′ |
|  | 5′-GCTCGCAGAGTCTCTGCACA-3′ |
| Gapdh | 5′-TGCGACTTCAACAGCAACTC-3′ |
|  | 5′-CTTGCTCAGTGTCCTTGCTG-3′ |
