# Supplementary figures and images for: Age-associated telomere shortening in mouse oocytes
Source: Reprod Biol Endocrinol. 2013 Nov 21;11:108. doi: 10.1186/1477-7827-11-108 (PMC3842639; doi:10.1186/1477-7827-11-108)

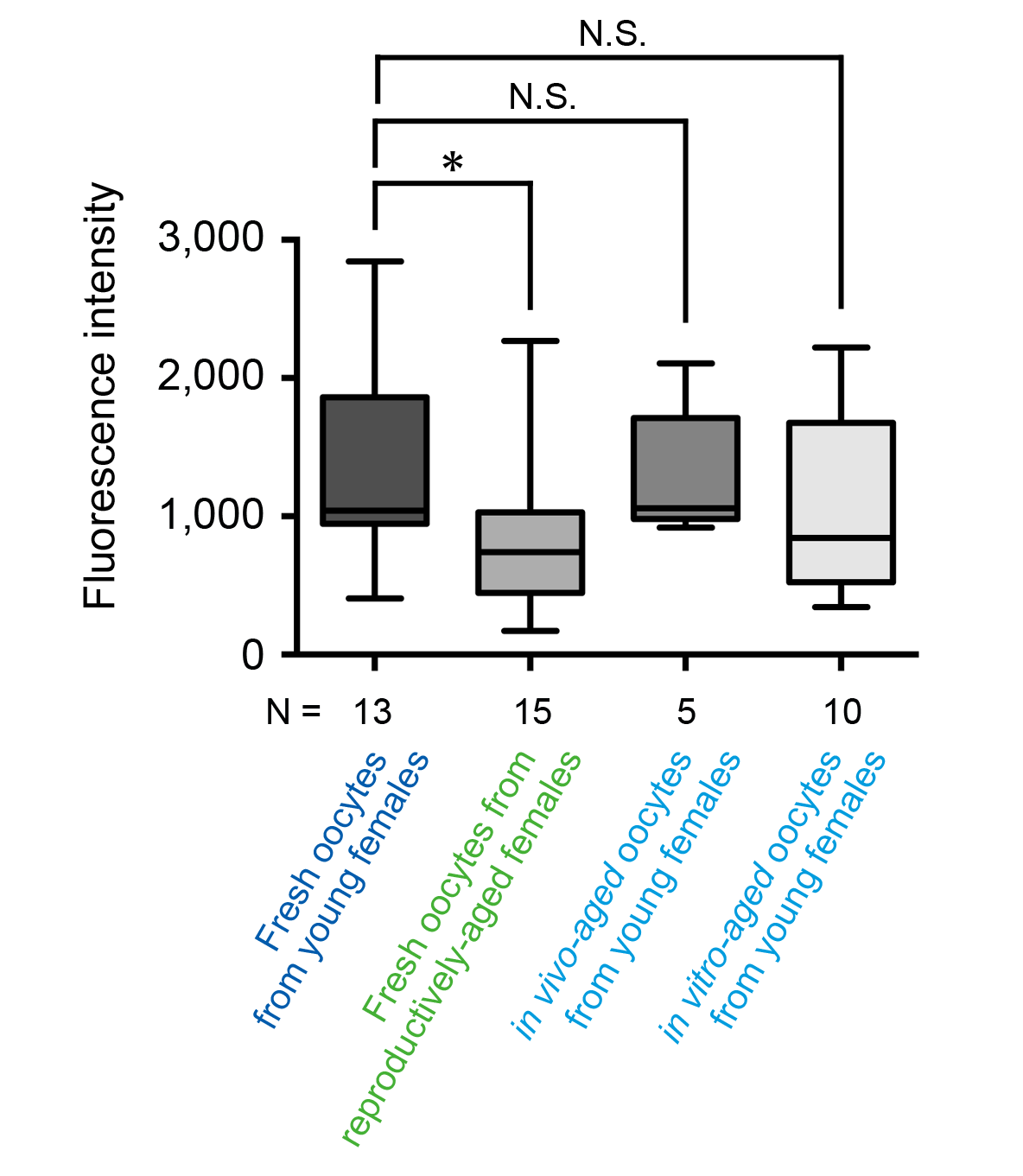

Supplement: Additional file 2: Figure S1 — Comparison of average telomere fluorescence of oocytes from young females, oocytes from reproductively-aged females, and in vivo-aged and in vitro-aged oocytes from young females. Box plots represent the median and inter-quartile ranges, with whiskers representing the 5th and 95th percentiles, respectively. *P < 0.05 (N: total number of oocytes). [file 1477-7827-11-108-S2.tiff]
